# A new risk score based on twelve hepatocellular carcinoma-specific gene expression can predict the patients’ prognosis

**DOI:** 10.18632/aging.101563

**Published:** 2018-09-21

**Authors:** Ting Lin, Jingxian Gu, Kai Qu, Xing Zhang, Xiaohua Ma, Runchen Miao, Xiaohong Xiang, Yunong Fu, Wenquan Niu, Junjun She, Chang Liu

**Affiliations:** 1 Department of Hepatobiliary Surgery, The First Affiliated Hospital of Xi’an Jiaotong University, Xi’an 710061, Shaanxi, China; 2 Institute of Clinical Medical Sciences, China-Japan Friendship Hospital, Beijing 100029, China; 3 Department of General Surgery, The First Affiliated Hospital of Xi’an Jiaotong University, Xi’an 710061, Shaanxi, China; * Equal contribution

**Keywords:** hepatocellular carcinoma, prognosis, tumor-specific genes, risk score

## Abstract

A large panel of molecular biomarkers have been identified to predict the prognosis of hepatocellular carcinoma (HCC), yet with limited clinical application due to difficult extrapolation. We here generated a genetic risk score system comprised of 12 HCC-specific genes to better predict the prognosis of HCC patients. Four genomics profiling datasets (GSE5851, GSE28691, GSE15765 and GSE14323) were searched to seek HCC-specific genes by comparisons between cancer samples and normal liver tissues and between different subtypes of hepatic neoplasms. Univariate survival analysis screened HCC-specific genes associated with overall survival (OS) in the training dataset for next-step risk model construction. The prognostic value of the constructed HCC risk score system was then validated in the TCGA dataset. Stratified analysis indicated this scoring system showed better performance in elderly male patients with HBV infection and preoperative lower levels of creatinine, alpha-fetoprotein and platelet and higher level of albumin. Functional annotation of this risk model in high-risk patients revealed that pathways associated with cell cycle, cell migration and inflammation were significantly enriched. In summary, our constructed HCC-specific gene risk model demonstrated robustness and potentiality in predicting the prognosis of HCC patients, especially among elderly male patients with HBV infection and relatively better general conditions.

## Introduction

Hepatocellular carcinoma (HCC) is the most common type of the liver cancer. The incidence and death rate of the liver cancer increase rapidly worldwide [1]. In China, the liver cancer remains the third cause of cancer-related deaths and the third commonly diagnosed cancer [2]. Some therapeutic strategies such as curative resection, liver transplantation, radiofrequency ablation (RFA) and transarterial embolization (TAE) may be promising for the treatment of HCC, whereas its prognosis remains unsatisfactory due to high recurrence and metastasis rates [3–6]. The underlying mechanisms may involve epigenetic alterations and genetic mutations, as well as lack of reliable gene signatures [7–10]. It is hence of clinical importance to construct a robust molecular model that can reliably predict the prognosis of HCC and has the potential to guide personalized therapeutic first-line treatment strategies.

Tremendous improvement has been made over the past decade in seeking prognostic genetic biomarkers for HCC [11]. In particular, the development of high-throughput platforms including DNA microarrays and RNA sequencing contributes greatly to the identification of potential biomarkers and therapeutic targets [12]. A large panel of genes have been reported to be associated with clinical outcomes of HCC, and some genetic prediction models have developed accordingly [13–16]. Besides, other types of genetic forms, such as long non-coding RNA (lncRNA) have been demonstrated to play a role in the pathogenesis of HCC [17]. However, few of these genetic biomarkers have been applied to clinical practice owing to lack of general extrapolation and experimental or clinical validation. Thus, identification of more reliable genetic biomarkers that can accurately predict HCC prognosis is still in the process of exploration and perfection.

Considering that HCC is an extremely heterogeneous disease and targeted therapies and personalized management become promising these days, the specific genomic sub-classification of HCC patients is of great significance for the improvement of prognosis assessment [18]. Here, we attempted to identify specific genes that are significantly dysregulated only in HCC rather than in the other subtypes of the liver cancer like intrahepatic cholangiocarcinoma (ICC) and the secondary liver cancer. Although therapeutic approaches for ICC are similar to HCC, the clinical outcome is relatively poorer than HCC, partly due to different molecular pathologic mechanisms [19,20]. As for the metastatic liver cancer (MLC), colorectal cancer liver metastasis (CRCLM) was adopted as an example here, which was a common type of secondary liver cancer [21]. Like other MLC, the treatment for CRCLM needs multidisciplinary approaches because of complex complications of primary cancer and secondary liver cancer [22–24]. Though hepatectomy is an optimal approach, the prognosis of CRCLM largely depended on primary cancer that differed from HCC [25–28]. Therefore, the specificity of the prognostic model is fetal to the precise prognostic prediction and therapeutic decision-making of HCC. In this study, we aimed to develop a specific, accurate and robust genetic prognostic risk score system for HCC.

## RESULTS

### Identification of HCC-specific gene list

**Figure 1:**
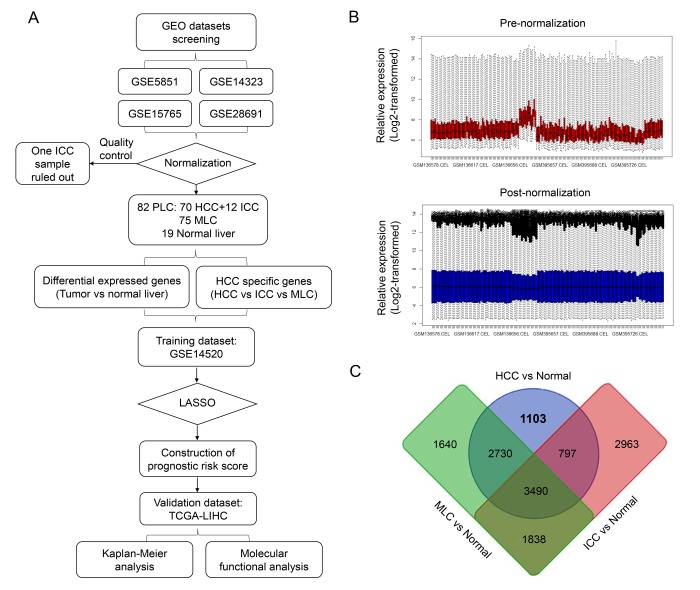
**Identification of HCC-specific gene list.** (**A**) Overview of the overall design and analytic procedure of the study. (**B**) Relative expression of all the included sample before (*Upper & Red*) and after (*Lower & blue*) RMA normalization. All the expression value was transformed by “log2()” algorism. (**C**) Venn diagram among three lists of dysregulated genes between three different subtypes of liver cancer (HCC, ICC and MLC) and normal liver. 1103 HCC-specific genes, 2963 ICC-specific genes and 1640 MLC-specific genes were generated through Venn selection.

**Figure 2:**
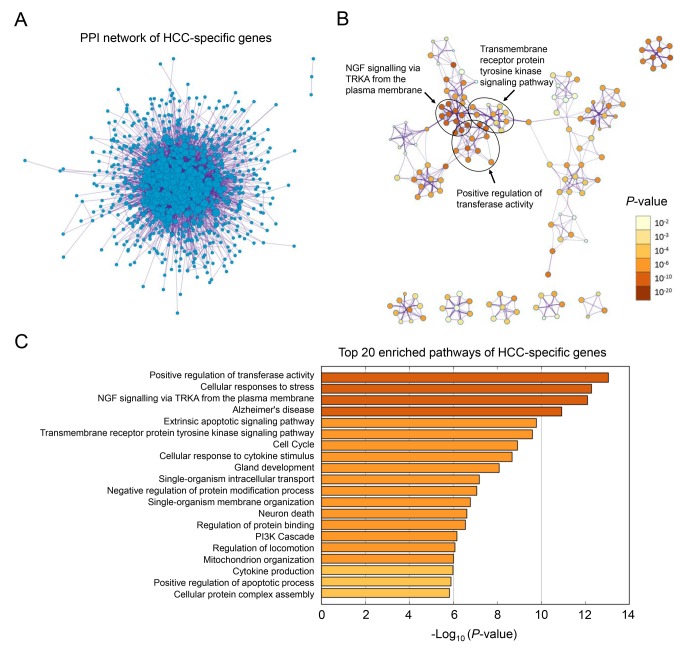
**Gene ontology analysis of HCC-specific genes.** (**A**) The PPI network of all the HCC-specific genes illustrated in Cytoscape. Each node represented a protein translated by an HCC-specific gene. (**B**) Network of 20 top-score modules (clusters) visualized in Cytoscape. Each cluster was made up of 10 best enriched GO terms within the threshold of Kappa-statistical similarity (0.3). Each node represented one enriched term and was colored by P value. In the figure, 3 representative pathways and the clusters they belonged to were marked. (**C**) The bar chart of 20 most enriched terms of HCC-specific genes arranged by -Log_10_ *P* value.

The overall workflow of this study is presented in Figure 1A. After background correcting, normalization and quality control of the selected raw profiles of four datasets (GSE5851, GSE28691, GSE15765 and GSE14323), 70 HCC samples, 75 CRCLM samples, 12 ICC samples and 19 normal control samples were obtained and considered eligible for further analysis. The relative expression of all samples pre- and post-normalization is shown in Figure 2B. Next, we compared three subtypes of the liver cancer samples with control samples, respectively. Three lists of dysregulated genes were identified and subjected to Venn selection for cancer-specific genes. A total of 1103 HCC-specific genes were identified including 816 up-regulated and 287 down-regulated (Figure 1C). The list of HCC-specific genes and their comparisons between HCC and non-tumor samples are shown in Supplementary Table S1. Moreover, gene ontology analysis of these genes revealed that “positive regulation of transferase activity”, “NGF signaling via TRKA from the plasma membrane”, and “transmembrane receptor protein tyrosine kinase signaling pathway” were significantly enriched biological processes and pathways which might be associated with HCC progression (Figure 2B). The protein-protein interaction (PPI) network of HCC-specific genes and top 20 significant enriched terms are presented in Figure 2A and Figure 2C.

### Construction of HCC-specific gene risk score system

**Figure 3:**
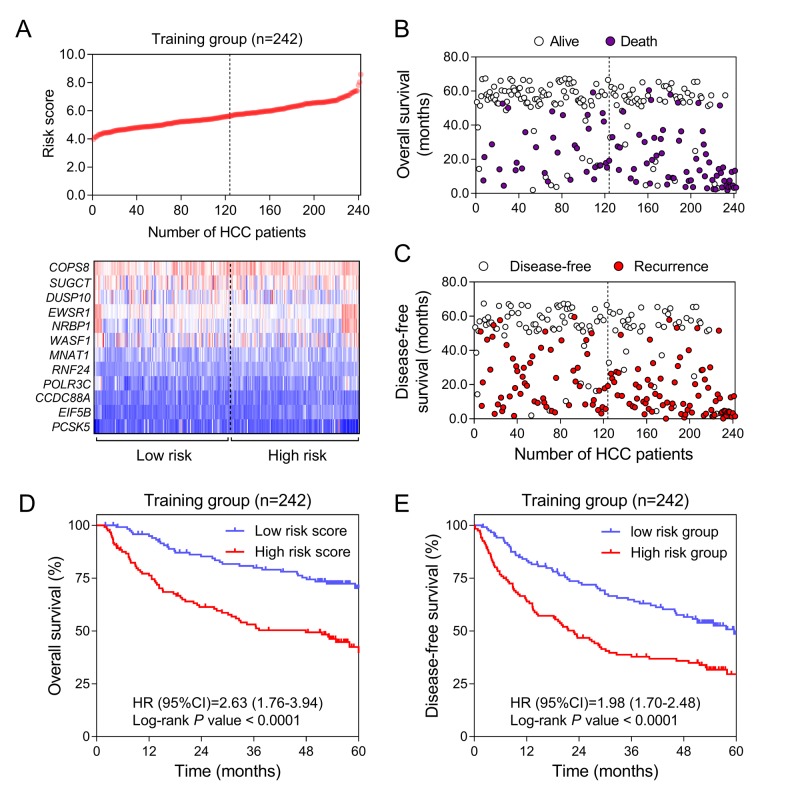
**Construction of HCC-specific gene risk score system using GSE14520.** (**A**) HCC-specific risk score analysis in GSE14520. (*Upper*) The distribution of the risk score of 242 included samples. (*Lower*) Heatmap of the expression value of each gene in HCC-specific gene signature corresponding to each patient above. Red: high expression; Blue: low expression. (**B** and **C**) Survival (**B**) and recurrence (**C**) status of every patient in the training dataset (N=242). (**D** and **E**) Kaplan-Meier curves to compare OS (**D**) and DFS (**E**) of high-risk and low-risk groups in GSE14520.

To explore the prognostic value of HCC-specific genes, univariate survival analysis by Cox proportional hazards models of each gene from the training data (GSE14520) was conducted. All the genes with significant *P* values were screened for next-step model construction. Eventually, the score formula comprised of 12 optimal genes was developed by LASSO: Risk score = 0.66 × (expression value of *RNF24*) + (-0.61) × (expression value of *COPS8*) + 0.40 × (expression value of *EWSR1*) + (-0.40) × (expression value of *SUGCT*) + 0.38 × (expression value of *PCSK5*) + 0.35 × (expression value of *POLR3C*) + 0.31 × (expression value of *NRBP1*) + 0.27 × (expression value of *MNAT1*) + 0.18 × (expression value of *EIF5B*) + (-0.15) × (expression value of *DUSP10*) + 0.08 × (expression value of *WASF1*) + 0.07 × (expression value of *CCDC88A*). In this risk score system, three genes (*COPS8*, *SUGCT* and *DUSP10*) were proved to be positively associated with OS, while nine of them (*EIF5B*, *MNAT1*, *WASF1*, *EWSR1*, *POLR3C*, *RNF24*, *PCSK5*, *NRBP1* and *CCDC88A*) were negatively related to OS according to the negativity or positivity of their coefficients. The contribution of each gene made to this risk score model was weighted by absolute value of coefficients. Every patient would get a risk score according to the expressions of the 12 HCC-specific genes of themselves (Figure 3A). This risk score was considered to correlate with the individual overall survival (OS). The median of all patients’ scores used as the cut-off value divided the whole group into the high-risk and the low-risk groups (Figure 3A). The OS and DFS status of each patient in the training dataset was shown in Figure 3B and Figure 3C. From Kaplan-Meier analysis of GSE14520, high-risk group was thought to be associated with poor prognosis while low-risk group was predicted to have the opposite outcome (Figure 3D & 3E).

### Validation and development of 12 HCC-specific gene signature for prognosis

**Figure 4:**
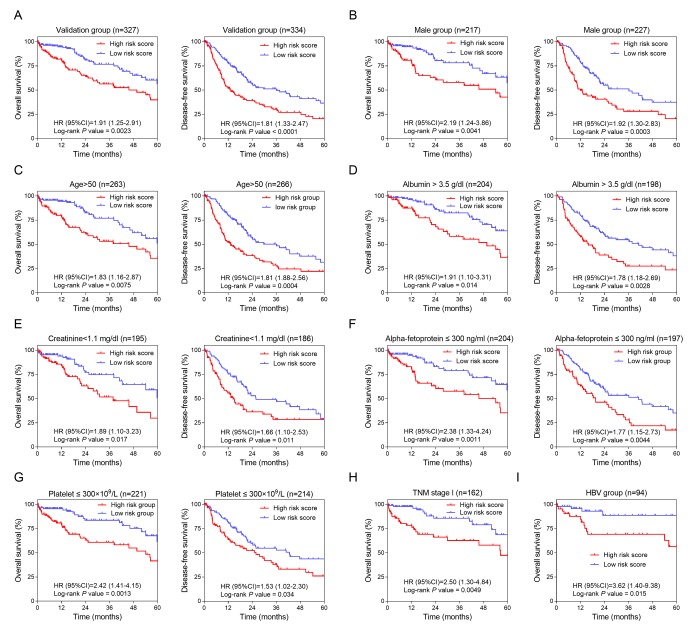
**Validation and development of HCC-specific risk score system.** (**A**) Kaplan-Meier curves of OS (*Left*) and DFS (*Right*) in the validation dataset. (**B**, **C**, **D**, **E**, **F** and **G**) Kaplan-Meier curves of OS (*Left*) and DFS (*Right*) in the subgroups stratified by gender (Male) (B), age (>50) (**C**), ALB (> 3.5 g/dl) (**D**), CRE (< 1.1 mg/dl) (**E**), AFP (≤ 300 ng/ml) (**F**) and PLT (≤ 300×10^9^/L) (**G**). (**H** and **I**) Kaplan-Meier curves of OS in the subgroups stratified by TNM stage (stage I) (**H**) and HBV infection (**I**).

To confirm the potentiality of the 12-HCC-specific gene prognostic model, Kaplan-Meier curve was performed to evaluate the association between the OS and DFS and our gene signature in validation dataset (TCGA) (Figure 4A). The cut-off values of TCGA cohort was 8.9. From the results, those high-risk patients had significantly shorter survival and earlier recurrence (*P* < 0.01).

In order to investigate the prognostic value of the risk score system in different patient groups with different characteristics, we firstly performed univariate and multivariate Cox regression analyses to confirm the relevance between different characteristics including the risk score and OS or DFS. From the Cox regression results, the risk score (> 8.9) and the race (THE White) were independent risk factors of OS (Table 1). And of DFS, the risk score (> 8.9), TNM stage (higher grade) and ECOG score (higher score) were independent risk factors (Table 2). Secondly, stratified analyses based on these clinical characteristics were carried out to identify the suitable patient groups of the risk score system (Table 3). The cut-off value (8.9) of each subgroup for survival analysis was consistent with the mother group. The stratified results of the validation dataset showed our HCC-specific gene signature was more applicable to the elderly male patients with preoperative relatively lower serum level of CRE (creatinine), AFP (alpha-fetoprotein) and PLT (platelet) and higher serum level of ALB (albumin) (Figure 4B, 4C, 4D, 4E, 4F and 4G). Besides, this risk score system exhibited better performance in predicting OS particularly in patients with HBV infection and in early stage (Figure 4H & 4I).

**Table 1:** Univariate/multivariate Cox regression analysis of clinicopathologic factors associated with OS in TCGA cohort.

**Variables**	**Univariate analysis**			**Multivariate analysis**	
	**HR (95% CI)**	***P* value**		**HR (95% CI)**	***P* value**
Risk score (> 8.9/≤ 8.9)	1.910(1.250-2.919)	0.003*		1.617(1.021-2.560)	0.040*
TNM stage (I/II/III/IV)	1.282(0.993-1.655)	0.057		—	—
Hepatitis (HBV/HCV/neither)	0.751(0.527-1.069)	0.112		—	—
Alcohol consumption (yes/no)	0.835(0.514-1.359)	0.469		—	—
Gender (female/male)	0.773(0.504-1.185)	0.237		—	—
Age (>50/≤50)	1.967(1.043-3.710)	0.037*		1.454(0.711-2.971)	0.305
Cirrhosis (yes/no)	0.865(0.471-1.587)	0.639		—	—
Albumin (≤3.5/>3.5 g/dl)	1.378(0.834-2.277)	0.211		—	—
Creatinine (<1.1/≥1.1 mg/dl)	0.739(0.455-1.199)	0.221		—	—
AFP ^a^ (≤300/>300 ng/ml)	0.900(0.513-1.582)	0.715		—	—
Platelet (≤300/>300×10^9^/L)	0.753(0.466-1.216)	0.246		—	—
Race (Asian/White)	0.760(0.638-0.904)	0.002*		0.766(0.630-0.930)	0.007*
BMI ^b^ (≥25<25)	1.028(0.650-1.626)	0.905		—	—
Family history (yes/no)	1.800(1.152-2.812)	0.010*		1.271(0.778-2.077)	0.339
ECOG ^c^	1.406(0.956-2.066)	0.083		—	—
Histological grade (G3-4/G1-2)	1.247(0.802-1.938)	0.327		—	—

Abbreviations: OS, overall survival; HR, hazard ratio; 95% CI, 95% confidence interval.

*: Statistically significant;

^a^: Alpha-fetoprotein;

^b^: body mass index;

^c^: Eastern Cooperative Oncology Group.

**Table 2:** Univariate/multivariate Cox regression analysis of clinicopathologic factors associated with DFS in TCGA cohort.

**Variables**	**Univariate analysis**			**Multivariate analysis**	
	**HR (95% CI)**	***P* value**		**HR (95% CI)**	***P* value**
Risk score (> 8.9/≤ 8.9)	1.841(1.358-2.494)	<0.001*		1.483(1.038-2.117)	0.030*
TNM stage (I/II/III/IV)	1.727(1.441-2.070)	<0.001*		1.568(1.274-1.929)	<0.001*
Hepatitis (HBV/HCV/neither)	0.943(0.760-1.170)	0.592		—	—
Alcohol consumption (yes/no)	1.061(0.767-1.468)	0.720		—	—
Gender (female/male)	0.982(0.711-1.355)	0.911		—	—
Age (> 50/≤ 50)	1.015(0.693-1.487)	0.940		—	—
Cirrhosis (yes/no)	1.271(0.861-1.877)	0.228		—	—
Albumin (≤ 3.5/> 3.5 g/dl)	1.033(0.702-1.519)	0.870		—	—
Creatinine (< 1.1/≥ 1.1 mg/dl)	0.739(0.511-1.069)	0.109		—	—
AFP ^a^ (≤ 300/> 300 ng/ml)	1.035(0.681-1.573)	0.873		—	—
Platelet (≤ 300/> 300×10^9^/L)	1.415(0.976-2.052)	0.067		—	—
Race (Asian/White)	0.787(0.575-1.078)	0.136		—	—
BMI ^b^ (≥ 25/< 25 kg/m^2^)	0.882(0.643-1.211)	0.437		—	—
Family history (yes/no)	0.920(0.655-1.292)	0.630		—	—
ECOG ^c^	1.697(1.406-2.049)	<0.001*		1.389(1.138-1.695)	0.001*
Histological grade (G3-4/G1-2)	1.186(0.867-1.621)	0.286		—	—

Abbreviations: DFS, disease-free survival; HR, hazard ratio; 95% CI, 95% confidence interval.

*: Statistically significant;

^a^: Alpha-fetoprotein;

^b^: body mass index;

^c^: Eastern Cooperative Oncology Group.

**Table 3:** Stratified analysis of overall and disease-free survival in TCGA samples.

**Characteristics**	**Overall survival**				**Disease-free survival**		
	**High-risk / low-risk**	**HR (95% CI)**	***P* value**		**High-risk / low-risk**	**HR (95% CI)**	***P* value**
Overall	154/173	1.905 (1.248-2.910)	0.0023*		164/170	1.811 (1.329-2.466)	<0.0001*
TNM stage							
Stage I	68/94	2.502 (1.295-4.835)	0.0049*		63/95	1.643 (0.9616-2.806)	0.0509
Stage II	36/38	1.383 (0.5167-3.700)	0.5161		39/37	1.098 (0.5908-2.041)	0.7632
Stage III	38/26	2.263 (0.9245-5.539)	0.0524		49/25	1.714 (0.9881-2.972)	0.0592
Hepatitis							
HBV	44/50	3.622 (1.398-9.384)	0.015*		44/48	1.736 (0.9153-3.294)	0.0873
HCV	25/29	2.661 (0.8091-8.750)	0.0644		23/28	1.263 (0.5953-2.682)	0.5234
Non-hepatitis	81/84	1.643 (0.9681-2.790)	0.0539		90/84	2.157 (1.421-3.276)	0.0002*
Alcohol consumption							
Yes	40/54	2.235 (0.8735-5.719)	0.0464*		49/58	3.069 (1.736-5.426)	<0.0001*
No	110/109	0.807 (1.107-2.951)	0.0188*		108/102	1.429 (0.9693-2.105)	0.0691
Gender							
Male	94/123	2.192 (1.243-3.863)	0.0041*		103/124	1.920 (1.304-2.826)	0.0003*
Female	60/50	1.420 (0.7451-2.704)	0.2847		61/46	1.711 (1.008-2.903)	0.048*
Age							
≤ 50	32/32	1.553 (0.4715-5.112)	0.4636		38/30	1.765 (0.8905-2.497)	0.0972
> 50	122/141	1.828 (1.165-2.868)	0.0075*		126/140	1.808 (1.278-2.558)	0.0004*
Cirrhosis							
Yes	34/42	3.445 (1.244-9.539)	0.0237*		33/42	0.9595 (0.522-1.764)	0.8938
No	51/77	1.323 (0.7002-2.500)	0.3701		52/74	1.866 (1.114-3.125)	0.0114*
Albumin (g/dl)							
≤ 3.5	44/38	2.379 (1.030-5.495)	0.061		41/36	1.203 (0.6193-2.337)	0.5811
> 3.5	87/117	1.911 (1.102-3.312)	0.0139*		82/116	1.784 (1.183-2.691)	0.0028*
Creatinine(mg/dl)							
< 1.1	87/108	1.888 (1.102-3.234)	0.0171*		81/105	1.665 (1.097-2.529)	0.0113*
≥ 1.1	47/48	2.148 (0.9684-4.765)	0.0507		44/45	1.428 (0.7646-2.668)	0.2451
Alpha-fetoprotein(ng/ml)							
≤ 300	79/125	2.379 (1.334-4.243)	0.0011*		76/121	1.770 (1.147-2.731)	0.0044*
> 300	45/18	2.148 (0.7583-6.082)	0.2203		41/17	1.425 (0.665-3.054)	0.3896
Platelet(×10^9^/L)							
≤ 300	101/120	2.421 (1.413-4.147)	0.0013*		95/119	1.527 (1.016-2.295)	0.0337*
> 300	34/38	1.338 (0.5988-2.988)	0.4481		31/34	1.772 (0.9258-3.391)	0.0671
Race							
Asian	65/63	4.354 (1.719-11.03)	0.0041*		83/65	2.046 (1.270-3.297)	0.0034*
White	76/95	1.612 (0.9556-2.720)	0.0623		69/91	1.901 (1.234-2.928)	0.0015*
BMI^a^							
< 25	78/71	2.331 (1.201-4.523)	0.0175*		93/71	1.472 (0.9494-2.281)	0.0855
≥ 25	62/91	1.858 (0.9573-3.608)	0.0454*		56/87	2.549 (1.546-4.204)	<0.0001*
Family history							
Yes	52/57	1.723 (0.9518-3.118)	0.0669		85/91	1.543 (0.8052-2.957)	0.1744
No	48/52	1.882 (1.073-3.301)	0.0214*		101/90	1.639 (1.099-2.446)	0.0135*
ECOG^b^							
=0	63/95	2.958 (1.476-5.931)	0.002*		59/93	1.463 (0.889-2.408)	0.1159
>0	47/52	1.454 (0.6708-3.150)	0.3143		62/52	2.165 (1.353-3.463)	0.001*
Histological grade							
G1/2	73/127	1.445 (0.805-2.596)	0.1908		82/126	1.936 (1.256-2.983)	0.0008*
G3/4	77/45	3.357 (1.698-6.636)	0.0042*		78/43	1.684 (1.033-2.747)	0.0457*

Abbreviations: HR, hazard ratio; 95% CI, 95% confidence interval.

*: Statistically significant;

^a^: body mass index;

^b^: Eastern Cooperative Oncology Group.

### Functional annotation of the established 12-gene signature

**Figure 5:**
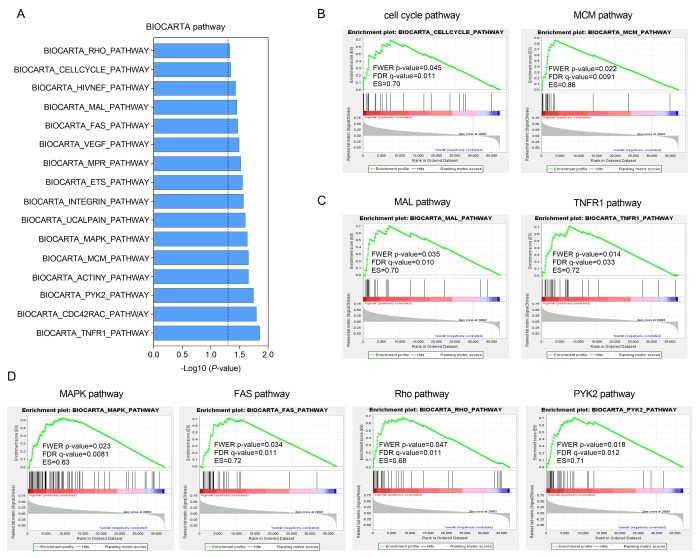
**Functional enrichment of HCC-specific gene signature in high-risk patients of TCGA series.** (**A**) The bar chart of 16 significantly enriched BIOCARTA pathways through GSEA. (**B**, **C** and **D**) Significantly enriched pathways associated with cell cycle (**B**), TNF-κB signaling (**C**) and MAPK pathway (**D**).

Gene set enrichment analysis (GSEA) was carried out in the high-risk group of the TCGA cohort to investigate key biological and cellular processes linked with poor prognosis. 16 in all significantly enriched BioCarta pathways are listed in Figure 5A. There were notable enriched KEGG pathways in high-risk patients included pathways connected with cell cycle like MCM pathway (Figure 5B), NF-κB signaling like MAL and TNFR1 pathways (Figure 5C) and classic MAPK-associated pathways such as FAS, Rho and PYK2 pathways (Figure 5D). These pathways mainly involved in cell proliferation and migration that might contributed to HCC metastasis and recurrence.

## DISCUSSION

In this study, we identified and validated a twelve-HCC-specific gene risk score system for predicting OS and DFS of HCC by multistep comparisons and screening. Firstly, to maintain the coincidence of all the datasets, we employed four mRNA expression profiles (GSE5851, GSE28691, GSE15765 and GSE14323) conducted by the same platform and integrated all datasets for normalization. After a comprehensive analysis, 1103 genes were selected as HCC-specific genes, among which 816 up-regulated genes were identified as risk factors, while 287 down-regulated as protective ones. In addition, pathway enrichment analysis revealed that these genes might influence cell cycle, migration and inflammation. Finally, 12-prognostic gene signature was constituted by LSAAO algorism and successfully validated in another independent dataset.

Of 12 identified genes in this prognostic model, three (*COPS8*, *SUGCT* and *DUSP10*) played a protective role in HCC patients, and by contrast the other nine (*EIF5B*, *MNAT1*, *WASF1*, *EWSR1*, *POLR3C*, *RNF24*, *PCSK5*, *NRBP1* and *CCDC88A*) acted as risk factors for HCC. *COPS8* (COP9 signalosome subunit 8) encodes a highly conserved protein complex that has been reported to be associated with gastric cancer and kidney cancer [29,30]. It has been proved in murine models that *COPS8* deficiency can impair ubiquitin-proteasome system (UPS) in the liver and the heart, respectively [31,32]. UPS is critical to protein degradation and homeostasis that is important to normal liver function [33]. *SUGCT* (succinyl-CoA: glutarate-CoA transferase) was demonstrated to be related to mitochondrial diseases and glutaric aciduria type III that might play a role in hepatic energetic metabolism [34–36]. *DUSP10* (dual specificity phosphatase 10) negatively regulate mitogen-activated protein kinase (MAPK) family, novel members of cellular proliferation and differentiation [37]. A recent study showed downregulation of *DUSP10* was associated with HCC metastasis [38]. *EIF5B*, one of eukaryotic translation initiation factors, was demonstrated to be involved in cell-cycle arrest in case of up-regulation [39,40]. *MNAT1*, a factor of the CDK-activating kinase (CAK) enzymatic complex, is vital to transcription. *MNAT1* was correlated with poor prognosis of several types of cancer including breast cancer, gastric cancer and colorectal cancer [41–43]. *WASF1*, also known as *WAVE1*, is a member of the Wiskott-Aldrich syndrome protein (WASP)-family highly expressed in brain and testis [44]. *EWSR1* (Ewing sarcoma breakpoint region 1 gene) has relation with gene expression, cell signaling and translocation, which is momentous to tumorigenesis [45]. There is evidence that *POLR3C* (RNA polymerase III subunit C) was be associated with virus infection [46]. *RNF24* (ring finger protein 24) encode a membrane protein that can interact with transient receptor potential cation channel subfamily C (TRPC) proteins [47]. The study by Wang et al. showed that *RNF24* correlated with the occurrence of esophageal adenocarcinoma [48]. *PCSK5* (proprotein convertase subtilisin/kexin type 5) was found to be dysregulated in different subtypes of triple-negative breast cancer (TNBC) [49]. A recent study by Bajikar et al. demonstrated *PCSK5* can inhibit TNBC metastasis by mediating retention of growth-differentiation factor 11 (GDF11) [50]. *NRBP1* (nuclear receptor binding protein 1) acts as a tumor suppressor and is commonly downregulated in a series of cancers such as breast cancer [51,52]. However, in prostate cancer, *NRBP1* was highly expressed and correlated with poor survival [53]. *CCDC88A* (coiled-coil domain containing 88A) regulates cytoskeleton remodeling and cell motility. Recent studies have suggested that *CCDC88A* played a role in metastasis and radio-resistance of HCC [54,55]. Of the 12 genes, 7 genes (*EIF5B*, *MNAT1*, *WASF1*, *POLR3C*, *RNF24*, *PCSK5* and *NRBP1*) were first reported to be associated with HCC prognosis in this study, which might give a few hints for future research into molecular mechanisms of HCC.

Gene enrichment analysis in the high-risk patients showed pathways involved with cell cycle, inflammation and migration were significantly enriched. Minichromosome maintenance (MCM) proteins are a group of ATPase, fundamental to the replication of DNA and the process of cell cycle [56]. Tumor necrosis factor receptor-1 (TNFR1) is the receptor of tumor necrosis factor (TNF), if activated, can cause proliferation or death of cells in different cellular context [57]. One of key roles of TNFR1 is to trigger NF-κB signaling by activating IκB kinase (IKK) complex [58]. MAL is an adaptor protein in the activation of Toll-like receptor 4 (TLR4)/ NF-κB pathway [59]. NF-κB is a nuclear transcription factor that acts as a regulator in various biological and pathological processes including inflammation, cell apoptosis, immune responses and tumorigenesis [60–62]. The mitogen-activated protein kinase/ extracellular signal-regulated (MAPK/ERK) pathway was a novel oncogenic pathway in most cancers [63]. MAPK mediates cellular apoptosis by Fas/FasL signaling pathways [64]. Rho is a kind of small GTP-binding protein triggering transduction of signaling cascades of MAPK pathways [65]. Proline-rich kinase-2 (Pyk2) is a non-receptor protein tyrosine kinase which participates in several pathways including MAPK and regulates cell proliferation, differentiation, adhesion and migration [66]. In a word, the findings of GSEA indicated that the HCC-specific gene signature might have potentials in the regulation of cell apoptosis, inflammatory responses, invasion and metastasis of HCC.

However, there are some limitations for this present study. First, the combination of samples for screening HCC-specific genes was small. Second, we constructed risk score system merely based on the gene expression levels, without considering the mutation, methylation, or other genetic events of genes that probably have an effect on the initiation and progression of cancer. Third, nearly 90% of patients in the discovery dataset had HBV, so the risk score system was established based on an HBV background. And further stratified analysis in validation cohort also demonstrated that this prognostic model was more applicable to HBV patients. Last but not the least, our HCC prognostic signature still needs to be validated in a larger population of patients from various backgrounds.

In conclusion, we constructed and confirmed an HCC-specific prognostic risk score system comprised of 12 genes. This risk score system could serve as a potential predictor for OS particularly in elderly male patients with HBV infection but in relatively better general conditions by risk-dependent stratification. From the results of functional annotations, pathways involved in cell cycle, NF-κB- and MAPK-associated pathways were significantly enriched, which might help better understand the molecular mechanisms underlying the initiation and progression of HCC. Moreover, our data provide new promising evidence on prediction biomarkers and targeted therapy for HCC.

## MATERIALS AND METHODS

### Microarray data collection and pre-processing

All microarray datasets were retrieved from the GEO database (https://www.ncbi.nlm.nih.gov/geo/) and the Cancer Genome Atlas (TCGA, http://cancergenome.nih.gov/) [67]. GSE5851, GSE28691, GSE15765 and GSE14323 from GEO were conducted through GPL571 (Affymetrix Human Genome U133A 2.0 Array). GPL571 platform was comprised of 22277 unique probes and tested more than 13500 genes (http://www.affymetrix.com/support/technical/byproduct.affx?product= hgu133-20). We selected 75 CRCLM samples from GSE5851 (61) and GSE28691 (14), 70 HCC samples and 13 ICC samples from GSE15765 and 19 normal liver tissue samples from GSE14323 for integrated normalization and analysis. One of ICC samples, GSM395714, was ruled out through quality control. GSE14520, as the training series downloaded from GEO database, included 247 HCC samples, 239 non-tumor tissue samples and 2 normal liver samples from healthy donors and was conducted by GPL571 and GPL3921 (Affymetrix HT Human Genome U133A Array), respectively. All the tumor samples (n=247) from GSE14520 were used as the training dataset. The raw fluorescence intensity profiles (*.CEL) of all the selected data from GEO were downloaded and normalized and further transformed to expression values through RMA algorism in the R environment (v3.4.3) [68]. 357 HCC samples from TCGA cohort were included as the validation group. The mRNA-seq data were preprocessed and submitted into analysis as the upper quantile normalized FPKM values.

### Screening of HCC-specific genes and gene ontology analysis

Differentially expressed genes (DEGs) between tumor groups (HCC, ICC and CRCLM) and normal group were obtained from GSE5851, GSE28691, GSE15765 and GSE14323. Only fold change (FC) ≥ 1.5 and *P* value for t-test < 0.05 were considered statistically significant. We then carried out Venn selection of cancer-specific genes among three DEG lists of the three types of hepatic neoplasms by Venny 2.1.0 (http://bioinfogp.cnb.csic.es/tools/venny/). Univariate survival analysis based on Cox proportional hazards of the HCC-specific genes was performed and genes with significant *P* values (< 0.05) from Log-rank tests were selected. Gene ontology (GO) analysis of HCC-specific genes was performed by Metascape, a user-friendly web tool for gene annotation and also a plugin of Cytoscape.

### LASSO statistical modeling

The 12-HCC-specific gene signature was derived from the least absolute shrinkage and selection operator (LASSO). LASSO is a linear regression algorism capable of variable selection and regularization simultaneously [69]. We carried out LASSO fitting method based on a series of λ using ‘glmnet’ package in the R environment (v3.4.3) [70]. The coefficients of each gene in risk score system were generated based on the expressions of each tissue sample in R studio at the same time.

### Confirmation and evaluation of risk score system

The HCC risk score model was validated and evaluated in TCGA cohort and the primary dataset (GSE14520). Univariate and multivariate Cox regression analyses were carried out with the validating series to estimate the association between various clinical characteristics containing the risk score and OS or DFS. Then stratified analysis based on clinical information was conducted in TCGA series. The median of TCGA cohort was accepted as optimal cut-off values to divide each group into the high-risk and the low-risk subgroups for survival analysis. All Kaplan-Meier curves were plotted and *P* values and hazard ratio (HR) with 95% confidence interval (CI) from log-rank tests were generated in GraphPad Prism 7.0.

### Gene set enrichment analysis

Gene set enrichment analysis (GSEA) was one of computational methods to identify significantly enriched biological processes and pathways. GSEA was carried out by the JAVA program (http://www.broadinstitute.org/gsea) based on Molecular Signature Database (MSigDB) [71]. Here, BioCarta (http://cgap.nci.nih.gov/Pathways/BioCarta_Pathways) pathway was enriched through GSEA in both high-risk and low-risk groups [72]. Each gene set would get an enrichment score (ES) that represented the number of overexpressed genes in this gene set. The false discovery rate (FDR) and the FWER *P* value of the gene sets < 0.05 were considered statistically significant.

### Statistical methods

Statistical analyses were performed using STATA/SE software (v12.0). False discovery rate (FDR) was applied to compare the expression of genes between tumor and non-tumor samples. FDR or *P* value < 0.05 was considered statistically different.

## Supplementary Material

Supplementary Table
