# Supplementary material for: A new risk score based on twelve hepatocellular carcinoma-specific gene expression can predict the patients’ prognosis
Source: Aging (Albany NY). 2018 Sep 21;10(9):2480–97. doi: 10.18632/aging.101563 (PMC6188480; doi:10.18632/aging.101563)
Supplement: Supplementary Table [file aging-10-101563-s001.pdf]

SUPPLEMENTARY TABLE

Table S1.

probeset ID	gene symbol	gene title	LogFC[a]	FDR[b]
202983_at	HLTF	helicase-like transcription factor	1.81	3.41E-29
210131_x_at	SDHC	succinate dehydrogenase complex, subunit C, integral membrane protein, 15kDa	1.44	2.14511E-26
212554_at	CAP2	CAP, adenylate cyclase-associated protein, 2 (yeast)	2.80	9.60115E-25
210417_s_at	PI4KB	phosphatidylinositol 4-kinase, catalytic, beta	1.32	6.89657E-24
32811_at	MYO1C	myosin IC	-0.89	1.70433E-23
219129_s_at	SAP30L	SAP30-like	1.36	2.14227E-22
202004_x_at	SDHC	succinate dehydrogenase complex, subunit C, integral membrane protein, 15kDa	1.17	2.75767E-22
221486_at	ENSA	endosulfine alpha	0.98	9.6368E-22
203715_at	TBCE	tubulin folding cofactor E	1.56	3.24734E-21
210460_s_at	PSMD4	proteasome (prosome, macropain) 26S subunit, non-ATPase, 4	1.46	4.38618E-21
39817_s_at	DNPH1	2'-deoxynucleoside 5'-phosphate N-hydrolase 1	1.04	4.38313E-21
216399_s_at	SCAPER	S-phase cyclin A-associated protein in the ER	1.01	1.04988E-20
203526_s_at	APC	adenomatous polyposis coli	1.20	9.10187E-20
221711_s_at	BABAM1	BRISC and BRCA1 A complex member 1	0.86	1.20476E-19
215150_at	YOD1	YOD1 deubiquitinase	1.15	1.55484E-19
204159_at	CDKN2C	cyclin-dependent kinase inhibitor 2C (p18, inhibits CDK4)	2.02	1.46634E-19
212853_at	DCUN1D4	DCN1, defective in cullin neddylation 1, domain containing 4	1.28	3.24184E-19
217782_s_at	GPS1	G protein pathway suppressor 1	0.95	3.07795E-19
221647_s_at	MIR6743 /// RIC8A	microRNA 6743 /// RIC8 guanine nucleotide exchange factor A	0.75	3.4838E-19
208938_at	PRCC	papillary renal cell carcinoma (translocation-associated)	1.17	1.25881E-18
201618_x_at	GPAA1	glycosylphosphatidylinositol anchor attachment 1	1.03	2.02464E-18
205655_at	MDM4	MDM4, p53 regulator	1.12	3.08052E-18
210360_s_at	MTSS1	metastasis suppressor 1	1.87	4.9371E-18
202916_s_at	FAM20B	family with sequence similarity 20, member B	1.09	4.7876E-18
203565_s_at	MNAT1	MNAT CDK-activating kinase assembly factor 1	0.81	4.82027E-18
201106_at	GPX4	glutathione peroxidase 4	0.86	1.28092E-17
211465_x_at	FUT6	fucosyltransferase 6 (alpha (1,3) fucosyltransferase)	-1.14	1.56646E-17
219311_at	CEP76	centrosomal protein 76kDa	1.05	1.84281E-17
217881_s_at	CDC27	cell division cycle 27	1.38	2.2452E-17
211251_x_at	NFYC	nuclear transcription factor Y, gamma	0.97	2.8815E-17
221703_at	BRIP1	BRCA1 interacting protein C-terminal helicase 1	0.99	4.29341E-17
214163_at	HSPB11	heat shock protein family B (small), member 11	1.13	4.23963E-17

222265_at	TNS4	tensin 4	-0.75	8.27649E-17
206474_at	CDK17	cyclin-dependent kinase 17	0.63	8.69675E-17
218909_at	RPS6KC1	ribosomal protein S6 kinase, 52kDa, polypeptide 1	1.76	1.04284E-16
218107_at	WDR26	WD repeat domain 26	1.08	1.10986E-16
209093_s_at	GBA /// GBAP1	glucosidase, beta, acid /// glucosidase, beta, acid pseudogene 1	1.72	1.10626E-16
207125_at	ZNF225	zinc finger protein 225	0.63	1.12671E-16
212570_at	ENDOD1	endonuclease domain containing 1	-0.84	1.2042E-16
210399_x_at	FUT6	fucosyltransferase 6 (alpha (1,3) fucosyltransferase)	-1.20	1.37322E-16
207304_at	ZNF45	zinc finger protein 45	1.11	1.53842E-16
208973_at	ERI3	ERI1 exoribonuclease family member 3	0.92	2.1801E-16
211060_x_at	GPAA1	glycosylphosphatidylinositol anchor attachment 1	0.95	2.55463E-16
218079_s_at	GGNBP2	gametogenetin binding protein 2	0.94	2.76087E-16
201754_at	COX6C	cytochrome c oxidase subunit VIc	0.76	3.31423E-16
207645_s_at	CHD1L	chromodomain helicase DNA binding protein 1-like	1.42	4.12203E-16
220003_at	LRRC36	leucine rich repeat containing 36	-0.79	5.90651E-16
212415_at	Septin-6	septin 6	1.79	8.02104E-16
212413_at	Septin-6	septin 6	1.23	1.05307E-15
204600_at	EPHB3	EPH receptor B3	-0.84	1.04527E-15
210875_s_at	LOC100996668 /// ZEB1	uncharacterized LOC100996668 /// zinc finger E-box binding homeobox 1	1.22	1.0885E-15
221078_s_at	CCDC88A	coiled-coil domain containing 88A	1.09	1.1472E-15
212487_at	GPATCH8	G patch domain containing 8	-0.79	1.39083E-15
202596_at	ENSA	endosulfine alpha	0.90	1.48188E-15
202374_s_at	AURKAPS1 /// RAB3GAP2	aurora kinase A pseudogene 1 /// RAB3 GTPase activating protein subunit 2 (non-catalytic)	0.97	1.70461E-15
202243_s_at	PSMB4	proteasome (prosome, macropain) subunit, beta type, 4	0.96	1.91236E-15
202585_s_at	NFX1	nuclear transcription factor, X-box binding 1	0.94	2.19399E-15
202839_s_at	NDUFB7	NADH dehydrogenase (ubiquinone) 1 beta subcomplex, 7, 18kDa	0.85	2.52093E-15
212839_s_at	TROVE2	TROVE domain family, member 2	0.60	2.50941E-15
204301_at	KBTBD11	kelch repeat and BTB (POZ) domain containing 11	-1.64	2.70468E-15
209177_at	NDUFAF3	NADH dehydrogenase (ubiquinone) complex I, assembly factor 3	0.70	2.76847E-15
216267_s_at	TMEM115	transmembrane protein 115	1.25	3.18831E-15
220642_x_at	GPR89A /// GPR89B	G protein-coupled receptor 89A /// G protein-coupled receptor 89B	1.14	3.46465E-15
203036_s_at	MTSS1	metastasis suppressor 1	1.19	3.43813E-15
215158_s_at	DEDD	death effector domain containing	0.81	3.43867E-15
216218_s_at	PLCL2	phospholipase C-like 2	1.17	3.65733E-15
204906_at	RPS6KA2	ribosomal protein S6 kinase, 90kDa, polypeptide 2	-0.82	3.87422E-15
213242_x_at	CEP170B	centrosomal protein 170B	-0.84	3.89443E-15
201771_at	SCAMP3	secretory carrier membrane protein 3	1.08	4.21174E-15

215773_x_at	PARP2	poly (ADP-ribose) polymerase 2	0.84	4.23472E-15
203515_s_at	PMVK	phosphomevalonate kinase	0.90	5.23647E-15
206206_at	CD180	CD180 molecule	-0.68	5.21883E-15
222045_s_at	PCIF1	PDX1 C-terminal inhibiting factor 1	0.76	5.28646E-15
211220_s_at	HSF2	heat shock transcription factor 2	0.61	5.96772E-15
37022_at	PRELP	proline/arginine-rich end leucine-rich repeat protein	-1.00	5.93428E-15
217847_s_at	THRAP3	thyroid hormone receptor associated protein 3	0.78	5.99118E-15
203013_at	ECD	ecdysoneless homolog (Drosophila)	0.94	6.23892E-15
205661_s_at	FLAD1	flavin adenine dinucleotide synthetase 1	1.36	8.53762E-15
210635_s_at	KLHL20	kelch-like family member 20	1.28	8.62145E-15
218037_at	FAM134A	family with sequence similarity 134, member A	0.93	1.20747E-14
216293_at	CLTA	clathrin, light chain A	-0.81	1.30517E-14
203556_at	ZHX2	zinc fingers and homeoboxes 2	1.01	1.84173E-14
210012_s_at	EWSR1	EWS RNA-binding protein 1	0.87	1.9622E-14
203776_at	GPKOW	G patch domain and KOW motifs	0.83	2.06291E-14
37577_at	ARHGAP19	Rho GTPase activating protein 19	0.79	2.47379E-14
215690_x_at	GPAA1	glycosylphosphatidylinositol anchor attachment 1	0.91	2.98627E-14
203454_s_at	ATOX1	antioxidant 1 copper chaperone	1.08	3.07285E-14
212092_at	PEG10	paternally expressed 10	3.54	3.55223E-14
214298_x_at	Septin-6	septin 6	1.37	3.82465E-14
209365_s_at	ECM1	extracellular matrix protein 1	-1.30	4.0429E-14
215420_at	IHH	indian hedgehog	-1.07	4.47E-14
215099_s_at	RXRB	retinoid X receptor, beta	0.97	4.5952E-14
207361_at	HBP1	HMG-box transcription factor 1	0.60	5.09087E-14
202752_x_at	SLC7A8	solute carrier family 7 (amino acid transporter light chain, L system), member 8	-0.91	5.90659E-14
211205_x_at	PIP5K1A	phosphatidylinositol-4-phosphate 5-kinase, type I, alpha	1.02	6.40865E-14
212640_at	PTPLB	protein tyrosine phosphatase-like (proline instead of catalytic arginine), member b	1.02	6.40152E-14
212159_x_at	AP2A2	adaptor-related protein complex 2, alpha 2 subunit	0.67	6.56532E-14
217932_at	MRPS7	mitochondrial ribosomal protein S7	0.81	7.18957E-14
200743_s_at	TPP1	tripeptidyl peptidase I	0.97	7.17219E-14
210706_s_at	RNF24	ring finger protein 24	-0.77	8.2877E-14

[a]: logFC was the logarithm Fold Change as HCC tissue being compared with non-tumor tissue. +/- represented up-/down- regulated expression level in HCC.

[b]: False discovery rate.
